# Recurrent Lower Respiratory Tract Infections Due to Mounier-Kuhn Syndrome

**DOI:** 10.7759/cureus.15437

**Published:** 2021-06-04

**Authors:** Collin J O'Bryan, Ronald Espinosa, Subramanyam Chittivelu, Vivian Wrenn

**Affiliations:** 1 Internal Medicine-Pulmonology, University of Illinois College of Medicine Peoria, Peoria, USA; 2 Pulmonary and Critical Care Medicine, University of Illinois College of Medicine Peoria, Peoria, USA; 3 Internal Medicine-Pediatrics, University of Illinois College of Medicine Peoria, Peoria, USA

**Keywords:** mounier-kuhn syndrome, tracheobronchomegaly, bronchiectasis, pulmonology, non-cf bronchiectasis

## Abstract

Mounier-Kuhn syndrome (MKS) is a rare disorder characterized by recurrent lower respiratory tract infections and bronchiectasis due to dilation of the trachea and bronchi. Diagnosis is made based on clinical suspicion along with radiographic evidence of tracheobronchomegaly. Mucolytic agents and chest physiotherapy have been shown to offer symptomatic improvement, and definitive surgical treatment is reserved for those with persistent symptoms. Herein, we report a case of MKS in a 72-year-old woman with bronchiectasis and recurrent multidrug-resistant lower respiratory tract infections.

## Introduction

Mounier-Kuhn syndrome (MKS) is a rare disorder characterized by dilation of the trachea and first to fourth-order bronchi secondary to atrophy of the muscular and elastic tissue [1]. Most cases are sporadic, although there are some reports of autosomal recessive transfer among families [2]. MKS is much more common among men with an 8:1 ratio of cases among men and women [3]. Loss of structural integrity of the airway in MKS results in tracheobronchomegaly and increased collapsibility, most prominent on expiration, leading to impaired clearance of respiratory secretions [4]. MKS typically manifests as recurrent lower respiratory tract infections and bronchiectasis, although hemoptysis, chronic cough, and dyspnea may also be seen [5]. Up to 7% of patients may present with severe tracheobronchomegaly, requiring mechanical ventilation [6]. Local tracheobronchomegaly may occur with prolonged endotracheal intubation, tracheostomy, or vascular rings; however, little is known about the etiology of the diffuse tracheobronchomegaly seen in MKS [6]. Histologically, loss of elastic fibers within the tracheal wall along with atrophy of airway smooth muscle is seen [4]. The diagnosis is made based on clinical findings and chest radiographs or CT showing a coronal tracheal diameter of >30 mm [3]. Other suggestive CT findings include diverticula from the weakness of the connective tissue along the length of the trachea [5]. To diagnose MKS, secondary causes of tracheobronchial enlargement must also be ruled out, such as Ehlers-Danlos syndrome, Marfan syndrome, and ataxia-telangiectasia [2]. Diagnosis is further supported by nonspecific findings, such as a plain chest radiograph showing a tracheal size exceeding the width of the vertebral columns and an obstructive pattern on pulmonary function tests (PFTs) [7,8]. The syndrome may also be evident on bronchoscopy, showing increased tracheal diameter and expiratory tracheal collapse; redundant tracheal tissue may even obstruct the bronchoscopic view [8].

Treatment strategies in MKS have not changed significantly since the syndrome was classified by Mounier-Kuhn in 1932 [4]. Treatment is largely focused on supportive care during exacerbations with few options available to directly target the anatomic airway abnormalities. Two procedures have been shown to be effective in stabilizing the airway in MKS: stents and surgical tracheobronchoplasty [4]. Stent placement stabilizes the weakened airway and is typically first-line to determine if patients would benefit from definitive surgical intervention [9]. Stent placement is challenging due to the large size of the stent required to stabilize the already enlarged airway and complications are common, such as stent migration and mucus plug impaction [9]. To reduce the risk of stent migration, the use of Y-type stents has been employed with improvements in outcomes [6]. Another approach is tracheobronchoplasty, which reestablishes the normal anatomy of the airway while maintaining mucociliary function [6]. In a retrospective study by Odell et al., they found that patients undergoing either of these two treatment options showed clinically significant improvements in the quality of life and symptoms, although no statistically significant changes were seen in PFTs [4].

## Case presentation

**Figure 1:**
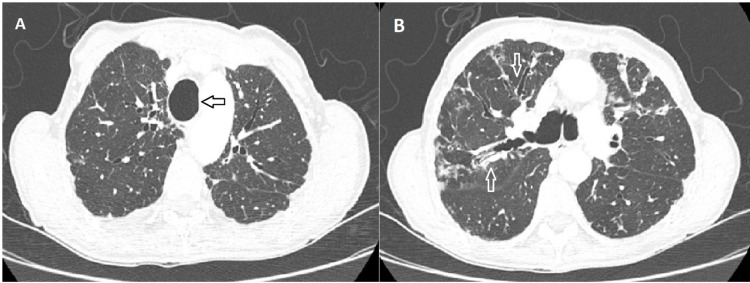
Axial HRCT images. (A) Axial HRCT showing a large tracheal size. (B) Axial HRCT showing bronchiectasis with dilated bronchi (arrows). HRCT: high resolution computed tomography

The patient was a 72-year-old woman with a past medical history of chronic cough with expectoration of thick sputum. She had been treated several times with antibiotics for chronic bronchitis. The patient did not have any history of childhood respiratory infections, tobacco smoking, aspiration pneumonia episodes, or neuromuscular disease. She was admitted to our facility because of fever, productive cough, wheezing, dyspnea, and hypoxemia with the diagnosis of lower respiratory tract infection. Pulmonary consultation was done to evaluate the patient for the management of chronic cough and dyspnea. Physical examination revealed pulmonary cachexia, bilateral coarse crackles, and wheezing. Digital clubbing was prominent in both hands. Fungal cultures and acid-fast bacilli smear were negative. Sputum cultures were positive for *Pseudomonas aeruginosa* and the patient was treated with broad-spectrum antibiotics. A chest CT scan was performed which revealed significantly enlarged trachea, bronchi, and bronchioles with mucoid impaction in distal airways (Figure 1). The coronal tracheal diameter was measured to be 32 mm. Her most recent PFTs were in 2016 which showed moderate obstructive lung disease with a forced expiratory volume in one second (FEV1) of 60% and an FEV1:FVC ratio of 71%. These features are consistent with recurrent pulmonary infection, leading to bronchiectasis and mucoid impaction due to MKS.

## Discussion

MKS is a rare disorder with an uncertain etiology that leads to the dilation of the trachea and first to fourth-order bronchi. The syndrome leads to bronchiectasis and recurrent respiratory infections due to enlarged airways and ineffective cough, leading to poor clearance of secretions [1]. While there is significant dilation of the airway during inspiration, the trachea and bronchi collapse during expiration, further impairing mucosal clearance [5]. Diverticula may also develop due to the weakened tissue, further contributing to the retention of respiratory secretions [7]. The diagnosis of MKS is made based on clinical and radiographic findings. Early diagnosis is rare as recurrent respiratory complaints are frequently initially attributed to chronic obstructive pulmonary disease (COPD), even when there is a lack of supporting evidence [8]. Treatment is dependent on severity and for most patients involves supportive measures, such as mucolytic agents and chest physiotherapy [3]. For patients with multiple tracheal diverticuli and fibrotic changes, definitive treatment targeting the anatomic cause of the disease with stent placement or tracheobronchoplasty may be performed [10]. Although studies have shown promising improvements in the quality of life and functional status after surgery, they have been limited by small sample sizes and poor long-term follow-up [4]. Longitudinal studies with larger study sizes must be conducted to assess the long-term benefits of these surgical measures.

We described a case of MKS in a woman with no smoking history or personal or family history of lung disease. This is a unique case as MKS has a strong male predominance and is typically seen among smokers [8]. A significant number of patients have also been diagnosed with COPD [11]. By identifying MKS as the etiology of our patient’s recurrent lower respiratory tract infections, a care plan can be established to determine the best route of care to maintain her functional status and prevent rehospitalizations.

## Conclusions

Recurrent lower respiratory tract infections and bronchiectasis with radiographic evidence of tracheobronchomegaly should raise suspicion for MKS. In those diagnosed with this rare condition, supportive treatment with mucolytic agents and chest physiotherapy should be offered to provide symptomatic relief and reduce the risks of recurrent lower respiratory tract infections. If symptoms are persistent and functional status is compromised, management targeting the underlying anatomic abnormalities with stent placement or tracheobronchoplasty should be considered.
